# Left ventricular ablation for ventricular tachycardia using a variable-loop circular pulsed field ablation catheter

**DOI:** 10.1007/s10840-026-02441-4

**Published:** 2026-09-19

**Authors:** Sania Jiwani, Frank Bogun, Thomas A. Franzon, Jackson J. Liang

**Affiliations:** 1 Section of Electrophysiology, Division of Cardiology, Department of Internal Medicine, University of Michigan, 1500 E Medical Center Dr, Ann Arbor, MI 48109 USA; 2 Department of Cardiology, Bronson Methodist Hospital, Kalamzoo, MI USA

**Keywords:** Pulsed field ablation, Ventricular Tachycardia, Retrograde aortic, Catheter Ablation

## Abstract

Pulsed field ablation (PFA) has been increasingly used for ventricular ablation. We present a novel case describing use of a variable-loop circular PFA catheter (Varipulse, Biosense Webster, Inc., Irvine, CA, USA) via a retrograde aortic approach for left ventricular ventricular tachycardia ablation. Retrograde aortic access was achieved using a long sheath (Ventrax system, Merit Medical, South Jordan, UT) permitting safe unsheathing and manipulation of the variable-loop PFA catheter to avoid entrapment with the mitral valve apparatus. The procedure was completed without complication and no VT recurrence was observed over 3 months follow-up. This case highlights the feasibility of this approach and expands the potential applications of this integrated PFA platform beyond its current indication for atrial fibrillation ablation.

**Supplementary Information:**

The online version contains supplementary material available at https://doi.org/10.1007/s10840-026-02441-4.

## Introduction

Ventricular tachycardia (VT) remains a major cause of morbidity and mortality, and catheter ablation has become an essential therapeutic strategy when antiarrhythmic drug therapy fails or is not tolerated. Although mapping technologies and ablation methods have improved considerably, radiofrequency ablation (RFA), the most widely used energy modality, remains constrained by several important shortcomings. Challenges such as inadequate lesion creation within dense scar tissue, the potential for unintended thermal damage to adjacent structures, and limited catheter stability in highly trabeculated ventricular areas continue to drive high recurrence rates. These challenges have motivated the search for alternative energy modalities that can achieve more effective and durable substrate modification while minimizing collateral damage.

Pulsed field ablation (PFA) is a largely nonthermal energy modality that induces irreversible electroporation through the delivery of microsecond-scale, high-voltage electrical fields, resulting in selective myocardial cell death with relative sparing of surrounding structures [1]. Clinical experience with PFA for VT remains in its early stages. We present, to our knowledge, the first reported case of the use of a variable-loop circular PFA catheter (Varipulse, Biosense Webster, Inc., Irvine, CA, USA) via a retrograde aortic approach for left ventricular (LV) VT ablation. This case highlights the feasibility of this approach and expands the potential applications of this integrated PFA platform beyond its current indication for atrial fibrillation ablation.

## Case description

**Fig. 1:**
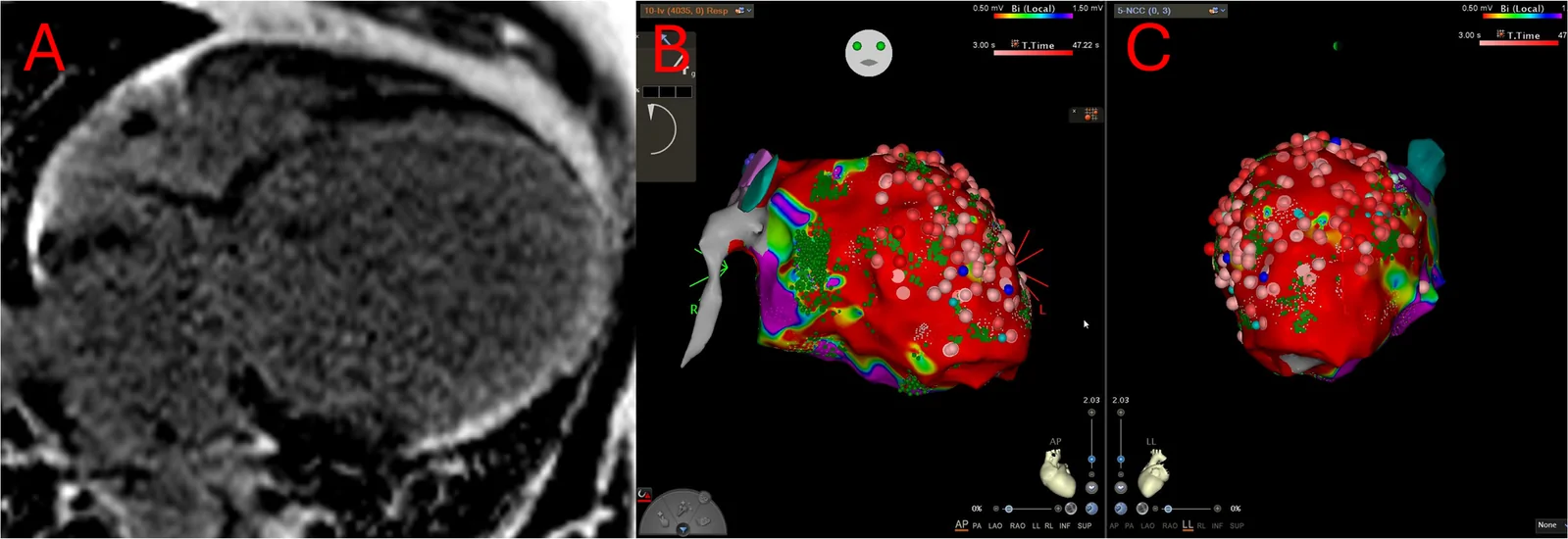
Panel **A** shows cardiac MRI (four chamber view) image showing extensive transmural LV scar secondary to prior LAD infarct. Electroanatomic maps (Anterior-posterior view in panel **B**, left lateral view in panel **C**) showing bipolar voltage and radiofrequency ablation lesions during initial ablation procedure

The patient is a 64-year-old male with prior LAD infarct, severe ischemic cardiomyopathy (LVEF < 20%, LV internal diameter 9.4 cm) and recurrent monomorphic VT requiring ICD therapies. CMR showed markedly dilated left ventricle and wall thinning with associated transmural late gadolinium enhancement involving the entire anterior, anterolateral and anteroseptal walls as well as the apex (Fig. 1, A). He had undergone VT ablation 2 months prior where multiple spontaneous non-clinical VTs occurred, with hemodynamic instability. Substrate modification was attempted, however, there was repeated RF-induced hemodynamically unstable VT requiring a total of 18 cardioversions. The procedure was terminated prematurely before completion of substrate modification given the patient’s tenuous hemodynamic status (Fig. 1, B and C). He had clinical recurrence of sustained VT 2 months later despite sotalol use and presented for repeat VT ablation. For the repeat procedure, we elected to use PFA to minimize the risk of RF-induced unstable VT.

After informed consent, the patient was brought to the EP laboratory. The procedure was performed under deep sedation (fentanyl, midazolam, propofol provided by certified registered nurse anesthetist). Ultrasound-guided femoral arterial and venous access was obtained. Heparin bolus and infusion were initiated to maintain an ACT > 350 s. An anatomic map of the ventricles and aortic cusps was created using a Soundstar ICE catheter and the CARTO mapping system (Biosense Webster, Irvine, CA, USA). Trans-septal catheterization was performed with a large curl steerable sheath (Agilis, Abbott, Abbott Park, IL) and a radiofrequency needle under ICE guidance. A multielectrode mapping catheter (Optrell FJ curve, Biosense Webster, Irvine, CA, USA) was advanced into the LV through the trans-septal Agilis sheath.

Voltage and activation mapping of the LV in paced rhythm was performed with the mapping catheter. Catheter ectopy induced sustained VT #1 – left bundle right inferior axis, negative precordial concordance, cycle length 280 msec (hemodynamically unstable requiring external cardioversion). Mapping the LV apex via transseptal approach was challenging due to the severely dilated LV and interaction with papillary muscles and chordae tendinae.

Due to anticipated difficulty with safely manipulating the variable-loop PFA catheter through the mitral valve, the decision was made to attempt retrograde aortic access instead. The right femoral arterial sheath was exchanged for a long sheath (MS Ventrax system, Merit Medical, South Jordan, UT) over a stiff wire. The aortic valve was crossed with the wire and pigtail sheath over which the sheath was advanced into the LV (Video 1). The mapping catheter was readvanced into the LV through the long sheath and further mapping of the LV was performed. The LV substrate was defined and areas of fractionated signal and late potentials were tagged.

**Fig. 2:**
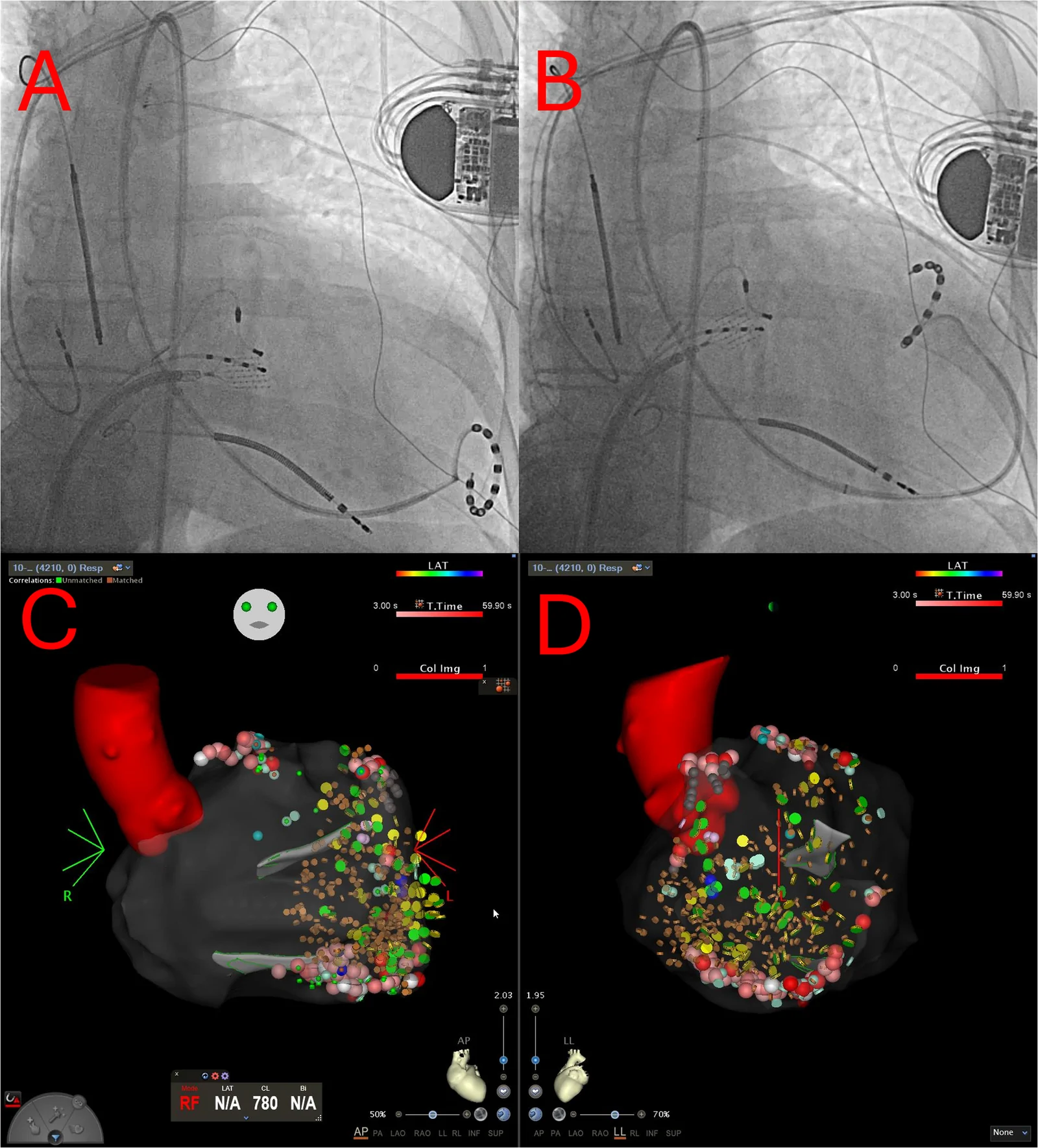
Fluoroscopic images (anteroposterior view) of variable-loop PFA catheter during ablation to the LV apex (Panel **A**) and LV anterior wall (Panel **B**) via a retrograde aortic approach. Electroanatomic maps of the left ventricle in anterior-posterior (Panel **C**) and left lateral (Panel **D**) views showing final PFA (brown dots) and RFA (red and pink dots) lesion set

Next, the Optrell catheter was exchanged for a variable-loop PFA catheter (Varipulse, Biosense Webster, Irvine, CA, USA) through the long sheath with the sheath tip positioned at the inferior LV, visualized on ICE and fluoroscopy. The variable-loop PFA catheter was unsheathed away from the mitral valve apparatus to prevent entanglement (Video 2). PFA applications were delivered to the apical LV scar aiming for scar homogenization. The variable-loop PFA catheter was unable to be visualized at the LV apex on CARTO system as it was outside the matrix due to massive LV size. Thus, ablation was guided by local signals, ICE, and fluoroscopy. Catheter manipulation induced sustained VT #2 – right bundle left superior axis, V2 transition. Mid-diastolic signals were noted on the variable-loop PFA catheter at the apex within the scar and delivered PFA applications here which resulted in clean termination of VT #2. A total of 28 PFA lesions were delivered within the apical scar aiming for scar homogenization and elimination of fractionated late potentials. Importantly, VT did not occur during PFA application (Fig. 2, A-D).

Due to difficulty maneuvering the variable-loop PFA catheter to target the inferior and septal aspects of the apical scar, the catheter was resheathed over the long sheath in the mid-LV and exchanged for a focal ablation catheter (QDOT MICRO, FJ curve, Biosense Webster, Irvine, CA, USA). Further empiric ablation was performed using radiofrequency energy at the border zones as well as the septal and inferior aspects of the apical scar. This was guided by local signals and pacemapping for VT1 and VT2. We also performed pacemapping for the clinical VT based on prior 12 lead EKG – right bundle right superior axis, V2 transition. The best pacematch for the clinical VT was noted at the lateral border of the apical scar. RFA was performed using 30 watts power and normal saline to homogenize the scar border zones and the areas that could not be reached with the variable-loop PFA catheter. There were no procedural complications, and the patient was discharged home 2 days later. The patient has not suffered any ICD shocks for 3 months since the procedure.

## Discussion

This case represents, to our knowledge, the first reported use of a variable-loop circular PFA catheter via a retrograde aortic approach for LV ventricular tachycardia ablation in a patient with ischemic cardiomyopathy.

PFA offers several mechanistic advantages compared to RFA for VT ablation in ICM. Because irreversible electroporation is a nonthermal phenomenon, the electric field is not impeded by collagen or fat. In a swine model of healed myocardial infarction, Younis et al. demonstrated that PFA produces uniform, well-demarcated lesions extending from the subendocardium through collagen and fat to the epicardial layers, achieving 72% transmurality compared to 30% with RFA (*p* ≤ 0.02) [2]. Im et al. similarly showed that PFA lesion depth in myocardial scar was significantly greater than RFA, and that surviving islands of myocardium within scar more than 10 mm from the catheter tip were also ablated - possibly because scar resistivity channels the PFA electric field toward myocardial cells [3]. Additionally, PFA maintains equivalent lesion depth when applied over chronic RF scars, making it potentially superior for redo VT ablation procedures [4]. 

The variable-loop circular PFA catheter is a smaller-caliber 8.5-French device with bidirectional deflection and a variable-loop design that allows adjustment of catheter diameter, potentially facilitating navigation within the LV cavity. Its full integration with the CARTO electroanatomic mapping system enables real-time high-density mapping and assessment of lesion formation. A critical challenge in ventricular PFA is limited catheter maneuverability restricting tissue apposition in certain regions of the LV. Fassini et al. noted that early loss of contact due to difficult catheter manipulation in the LV caused insufficient contact force and inadequate energy penetration in one of their two ICM VT cases treated with the pentaspline PFA catheter (FaraWave, Boston Scientific, Marlborough, MA, USA) [5]. The variable-loop PFA catheter’s smaller profile, bidirectional deflection and variable-loop diameter may partially address these limitations. However, the severe LV enlargement in our patient impeded adequate reach with the variable-loop PFA catheter as well as its visualization on the mapping system and additional RFA with a focal ablation catheter was required.

Importantly, the variable-loop circular design of the PFA catheter is comparable that of circular mapping catheters and prior reports have described catheter entrapment in the mitral valve apparatus requiring emergency surgery [6, 7]. Keeping this in mind, a retrograde aortic approach may be more suitable for ventricular ablation with the variable-loop PFA catheter in some situations, and the use of a long sheath advanced deep into the left ventricle permitting may permit safer unsheathing/resheathing and manipulation of the variable-loop catheter (under ICE and fluoroscopic guidance). Our case builds upon a report of retrograde aortic approach for PFA of VT by Martin et al., who used a pentaspline PFA catheter to successfully ablate an intramural VT circuit beneath the aortic valve that had failed prior conventional ablation [8]. 

At the time of the procedure, the PFA options available for clinical use at our institution were the pentaspline PFA catheter and variable-loop PFA catheter, and we were unable to obtain compassionate care use with the lattice-tip or focal dual energy ablation catheters due to clinical urgency of procedure. We decided to use the variable loop catheter due to the deflectability of the catheter in order to reach different regions of the LV (including the anterior wall) which would likely have been difficult to reach with a nondeflectable pentaspline PFA catheter in a patient with such a large LV, and also to facilitate more seamless integration to the eletroanatomic mapping system. The present case extends this experience to a different PFA platform with distinct catheter design characteristics and full integration with electroanatomic mapping. The options for PFA to facilitate VT ablation continues to expand and the advent of new catheters such as the lattice-tip dual energy catheter and other focal dual energy contact-force sensing catheters will allow for seamless integration with available mapping systems.

## Conclusion

This case demonstrates the feasibility of using a variable-loop circular PFA catheter via a retrograde aortic approach for scar-related VT ablation in ischemic cardiomyopathy - the first such report to our knowledge. Full integration with the mapping system and deflectable nature of this catheter may offer favorable workflow advantages. Prospective studies with longer follow-up are needed to determine whether the theoretical advantages of PFA over RF in ischemic scar translate into improved clinical outcomes, and to define the optimal catheter design, energy parameters, and access strategy for ventricular PFA.

## Supplementary Information

Below is the link to the electronic supplementary material.

Supplementary Material 1

Supplementary Material 2

## Data Availability

No datasets were generated or analysed during the current study.
